# Building resource constraints and feasibility considerations in mathematical models for infectious disease: A systematic literature review

**DOI:** 10.1016/j.epidem.2021.100450

**Published:** 2021-06

**Authors:** Fiammetta M. Bozzani, Anna Vassall, Gabriela B. Gomez

**Affiliations:** London School of Hygiene & Tropical Medicine, Department of Global Health and Development, 15-17 Tavistock Place, London, WC1H 9SH, United Kingdom

**Keywords:** Health system, Constraints, Mathematical models, Infectious disease, Systematic review

## Abstract

Priority setting for infectious disease control is increasingly concerned with physical input constraints and other real-world restrictions on implementation and on the decision process. These health system constraints determine the ‘feasibility’ of interventions and hence impact. However, considering them within mathematical models places additional demands on model structure and relies on data availability. This review aims to provide an overview of published methods for considering constraints in mathematical models of infectious disease.

We systematically searched the literature to identify studies employing dynamic transmission models to assess interventions in any infectious disease and geographical area that included non-financial constraints to implementation. Information was extracted on the types of constraints considered and how these were identified and characterised, as well as on the model structures and techniques for incorporating the constraints.

A total of 36 studies were retained for analysis. While most dynamic transmission models identified were deterministic compartmental models, stochastic models and agent-based simulations were also successfully used for assessing the effects of non-financial constraints on priority setting. Studies aimed to assess reductions in intervention coverage (and programme costs) as a result of constraints preventing successful roll-out and scale-up, and/or to calculate costs and resources needed to relax these constraints and achieve desired coverage levels. We identified three approaches for incorporating constraints within the analyses: (i) estimation within the disease transmission model; (ii) linking disease transmission and health system models; (iii) optimising under constraints (other than the budget).

The review highlighted the viability of expanding model-based priority setting to consider health system constraints. We show strengths and limitations in current approaches to identify and quantify locally-relevant constraints, ranging from simple assumptions to structured elicitation and operational models. Overall, there is a clear need for transparency in the way feasibility is defined as a decision criteria for its systematic operationalisation within models.

## Highlights

• Mathematical model capabilities to explore complex systems now enable priority-setting to consider local resource constraints.
• Common objectives of model-based analyses incorporating constraints are to assess real-world feasibility or allocate resources efficiently.
• Constraints may be incorporated via (i) model-based estimation; (ii) linkage of mathematical and health system models; or (iii) optimisation.
• Models can then project constrained intervention effects and costs and resource requirement s for delivering interventions at full scale.
• 'Health system constraints' should be systematically defined for routine operationalisation in model-based priority-setting.

## 1 Introduction

The launch of the Sustainable Development Goals, with their focus on Universal Health Coverage, has accelerated a shift in priority setting for health care interventions. The traditional focus on comparing the incremental cost-effectiveness of finite sets of interventions is being complemented with ranking and optimisation exercises across diseases and, in some cases, the whole health sector. Examples include defining essential benefits packages, disease-specific strategic plans and national health insurance coverage schemes for expanding access to health care and avoiding catastrophic costs for patients and households (Jamison et al., 2018). At the same time, it is being increasingly recognised that priority setting should take into account a range of non-financial constraints in any given setting and intervention area (Vassall et al., 2016) while considering multiple objectives alongside efficiency and effectiveness, such as equity and social protection.

Traditionally, the health care budget is the sole constraint considered in resource allocation models. However, policy-makers contend with several other constraints affecting feasibility *of implementation*, both on the supply (health system) and demand (patient) sides, when selecting interventions. These constraints may limit the pace of intervention scale-up (e.g. human resources scarcity in the short run); may be insurmountable even with increased resourcing (e.g. prioritisation of specific population groups, or an ethical obligation to provide treatment to all those in need); or may incur costs that are not observable when interventions are tested in research settings. Failure to account for such setting- and intervention-specific influences on the priority setting process itself and on the implementation of the resulting recommendations can result in unfeasible health interventions being recommended and, ultimately, in evidence being disregarded by decision-makers (Hauck et al., 2016; Mikkelsen et al., 2017).

**Fig. 1:**
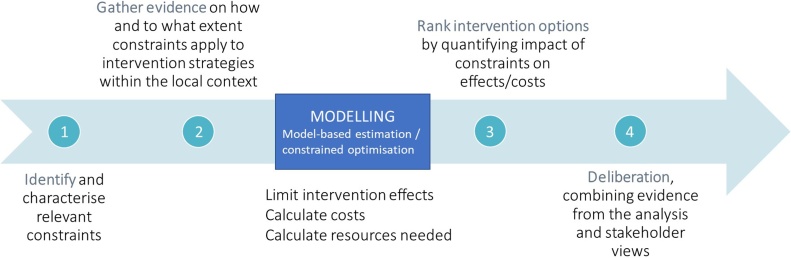
Framework for incorporating health system constraints in priority setting.

Mathematical models exploring complex systems have made a vital contribution to advancements in priority setting for infectious diseases. The recent development of user-friendly dynamic transmission models to prioritise new health technologies for infectious disease control increasingly allows policy-makers to account for setting-specific variations in factors such as epidemiological characteristics and input types and prices (Houben et al., 2016; Lubell et al., 2008; Stegmuller et al., 2017). Moreover, model-based priority setting may allow analysts to consider other country- and intervention-specific non-financial constraints that bind resource allocation decisions. For example, while transmission modelling analyses recommend intensified screening of all clinic patients for reaching the End TB Strategy targets in South Africa, this intervention is highly human resource (HR) intensive and increases the use of diagnostics downstream in the tuberculosis (TB) care cascade (Menzies et al., 2016). Thus, it might be a sub-optimal option compared to others in the TB portfolio when constraints on these inputs are taken into account. In this example, the effect of the constraints on intervention impact is parametrised in the model through changes in the rates of transitions between different compartments or states (the example of human resource constrains for TB care in South Africa is illustrated graphically in the Supplementary File 1 (Fig. 1A)). However, this may not be the only existing approach to the inclusion of constraints in these analyses.

The aim of this review is to establish how locally relevant non-financial constraints have been incorporated in model-based impact and cost-effectiveness analyses of infectious disease control interventions. In particular, we describe the constraints considered and how these were characterised and quantified in the models. Ultimately, we aim to discuss suitable model structures and techniques for implementing the constraints within them.

## 2 Materials and methods

A systematic search of the published literature was conducted to identify studies published before November 2020, that employ dynamic transmission models to assess infection control interventions in any disease and geographical area and that consider non-financial constraints to implementation. Preferred Reporting Items for Systematic Reviews and Meta-Analysis (PRISMA) statement and checklist (Liberati et al., 2009).

### 2.1 Search strategy

The MEDLINE and Embase databases were searched via the OvidSP platform for English language, full text studies on human subjects. The Scopus database was also searched without imposing any limits. The search strategy combined keywords on infectious diseases, dynamic transmission modelling, economic evaluation, priority setting and health systems research, including constraints and feasibility of health interventions. The following Medical Subject Heading (MeSH) terms were ‘exploded’ in MEDLINE and Embase: “Infectious Disease Transmission”, “Public Health Systems Research”, “Systems Analysis”, “Theoretical Models”, “Economic Models”, “Decision Support Techniques”. The full search strategy for each database and number of records retrieved (with and without limits, where applicable) are presented in Supplementary File 1B. A hand search of the reference lists of retained articles was also conducted to identify other potentially relevant literature.

### 2.2 Screening, data extraction and analysis

Search results were exported to EndNote (v. × 8) to eliminate duplicates. The abstract and titles of all unique records were then screened and articles were further excluded based on the following criteria: (i) language other than English; (ii) topic not related to human health; (iii) no reference to the application of health system constraints and infectious disease models; (iv) ineligible article type (clinical and/or pragmatic trials, feasibility or pilot or demonstration studies, editorials, conference proceedings, comments, letters and notes). The full texts of remaining articles were then reviewed and retained if they made reference to a formal method of applying non-financial constraints in priority setting using a mathematical model of infectious disease transmission. Articles using ‘static’ mathematical models or other model types and those that did not consider any constraints other than the budget or financial constraint were discarded.

Data was extracted from the retained records in the following categories: geographical and disease area of interest, type of intervention and level of the health system at which implementation occurred, transmission model structure, model population and projection timeframe, presence and type of economic analysis (including optimisation under a budget constraint), demand- and supply-side non-financial constraints considered as well as methods for identifying and quantifying the constraints, aim of the modelling exercise and formal method of incorporating the constraints in the analysis. The data was summarised using descriptive statistics and a thematic analysis of the contents of the articles was carried out to answer the study question.

For characterising how health system constraints were incorporated in models we drew on the work of Vassall and colleagues, who distinguished between proximal constraints, such as HR and pharmaceutical shortages, and distal constraints, such as cultural norms, values and regulations (Vassall et al., 2016). We then described how these constraints were analysed at different stages in the priority setting process using the framework shown in Fig. 1. Steps 1 and 2 refer to the identification and characterisation of health system constraints that apply to the intervention of interest in the specific context; steps 3 and 4 refer to the assessment of the constraints’ impact on intervention effects and/or costs, and to how this evidence is used in the deliberation process, highlighting how the views of stakeholders may still play a role alongside the quantitative evidence from modelling.

## 3 Results

**Fig. 2:**
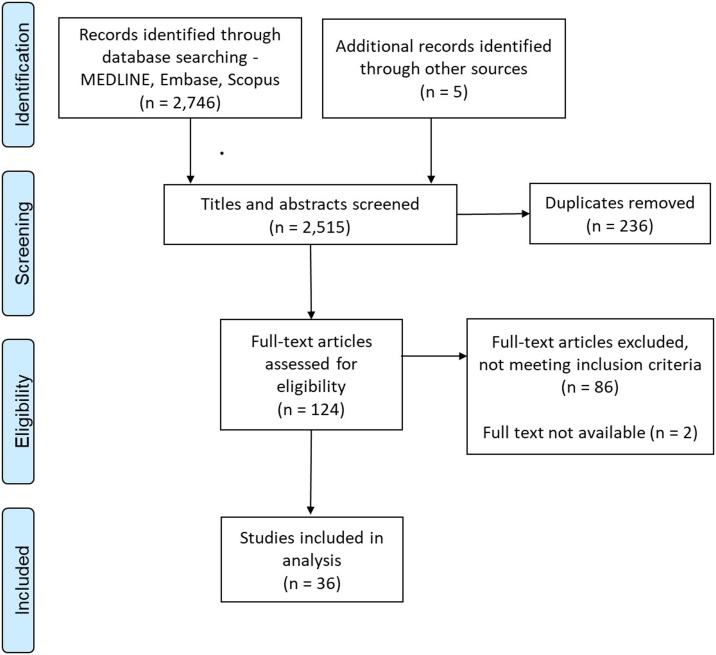
Flow chart of screening and selection process.

We identified 2751 unique citations, of which approximately one in 20 were eligible for full text screening. The PRISMA flow chart with details of the study screening and selection process is shown in Fig. 2.

After the selection process was completed, 36 studies were retained for analysis. The study characteristics, aims and model structures of all selected papers are summarised in Table 1. Approximately one third of the studies focused on a single country, predominantly in the low- and middle-income group, while eight studies were global in focus and a further three regional (two from sub-Saharan Africa and one from South-East Asia). Another seven studies, mostly from high-income settings, looked at one single municipality or health facility within a country. The disease area most represented in the literature was pandemic influenza, followed by human immunodeficiency virus (HIV) and TB.

**Table 1:** Study characteristics and mathematical models structure.

Lead author (year)	Setting	Disease area	Intervention	Level of health system	Study aim	Transmission model structure	Economic analysis
Adisasmito et al. (2015)	Local - Bali, Indonesia	Influenza	Pandemic influenza case management capabilities strengthening	Decentralised	Simulate influenza spread at the district level given existing resource gaps to inform preparedness planning	Density-dependent deterministic compartmental model (SEAIR)	–
Alistar et al. (2013)	Country - not specified	HIV	Multiple, user-defined HIV control interventions	National	Develop a model to guide setting-specific resource allocation across interventions along the HIV cascade	Frequency-dependent deterministic compartmental model (HIV disease stages and treatment status)	Cost-effectiveness analysis
Anderson et al. (2014, 2018)	Country - Kenya	HIV	Combination prevention interventions	Decentralised	Model the effect of prioritising key population and of short-term funding cycles on HIV prevention	Frequency-dependent deterministic compartmental model (susceptible, acute-, latent infection, pre-AIDS, AIDS)	Cost analysis
Bärnighausen et al. (2016)	Country - South Africa	HIV	Treatment as prevention (TaSP)	National	Model the effects of TaSP on universal ART coverage	Frequency-dependent deterministic compartmental model (susceptible, HIV infection stages)	–
Barker et al. (2017)	Regional - sub-Saharan Africa	HIV	ART differentiated care models	National	Model efficiency gains from different service delivery options	Frequency-dependent deterministic compartmental model (AIDS Impact Model, Estimation Projection Package)	Cost analysis
Bottcher et al. (2015)	Global	Influenza	Epidemic preparedness	National	Investigate the effects of disease-induced resource constraints on epidemic spreading	Density-dependent deterministic compartmental model (bSIS, recovery rate mediated by resources availability)	–
Bozzani et al. (2018, 2020), Sumner et al. (2019)	Country - South Africa	TB	Changes to screening and diagnostic algorithm	National	Develop a pragmatic approach for empirical estimation of health system constraints from routine data to parametrise models	Density-dependent deterministic compartmental model (susceptible, latent infection, active disease)	Cost and cost-effectiveness analysis
Chen et al. (2019)	Country – not specified	Sexually transmitted infection epidemic	Epidemic control	National	Model the effects of resource availability on rate of infection	Frequency-dependent deterministic compartmental model (SIS, recovery rate mediated by resources availability)	–
Cruz-Aponte et al. (2011)	Global	Influenza	Flu vaccination campaign during outbreak	National	Develop an accurate model of vaccine stockpiles for epidemic preparedness	Density-dependent deterministic compartmental model (SIR-like model including vaccines supply and numbers vaccinated)	–
Curran et al. (2016)	Global	General epidemic outbreak	Surge capacity planning	National	Develop a conceptual framework for integrating big data analytics with simulation, to provide real-time analysis of health system capacity during epidemics	Density-dependent deterministic compartmental model (SEIR)	–
Dalgiç et al. (2017)	Local - Seattle, US	Influenza	Flu vaccination campaign during outbreak	National	Compare age-specific vaccination strategies derived from agent-based simulation and from a deterministic compartmental model	Agent-based simulation and density-dependent deterministic compartmental model (SEIR), enhanced with mesh-adaptive direct search (MADS) algorithm to iteratively improve intervention strategies	Cost analysis
Ferrer et al. (2014)	Local - France	All-cause ICU visits	Strategies to cope with nurses shortages	Service	Explore impact of management strategies against nurse shortages on pathogen transmission within the ICU	Agent-based simulation	–
Hecht and Gandhi (2008)	Global	HIV	AIDS vaccination	National	Model determinants of demand, uptake dynamics and potential revenues from vaccine candidates	Discrete deterministic linear predictive model (vaccinated are a fraction of population in need dynamically estimated based on numbers of susceptibles who have access given constraints)	Cost analysis
Hontelez et al. (2016)	Regional - sub-Saharan Africa	HIV	ART scale-up (changing eligibility thresholds)	National	Model resource requirements to achieve ART coverage targets	Agent-based simulation	Cost-effectiveness analysis
Krumkamp et al. (2011)	Country - Thailand	Influenza	Epidemic preparedness	Decentralised	Simulate characteristics of an influenza outbreak and identify resource needs and gaps	Density-dependent deterministic compartmental model (SEAIR)	–
Langley et al., 2014; Lin et al. (2011)	Country - Tanzania	TB	New diagnostic technologies for parasitic disease	National	Model intervention effects on operational performance of the health system to accurately assess impact and cost-effectiveness	Deterministic compartmental model (SIR-like). Active diseases states of the model are expanded to include pathway from onset to diagnosis and linkage to treatment from operational model	Cost-effectiveness analysis
Marks et al. (2017)	Global	Yaws	Eradication campaign (mass azythromycin treatment followed by case finding and targeted treatment)	National	Determine the feasibility and optimal strategy for yaws eradication	Stochastic compartmental model (Markov model with susceptibles and primary, latent and secondary infection)	–
Martin et al. (2015a, b)	Local - New York state, US	HIV	Policy change to increase HIV testing and linkage to care	Decentralised	Assess health outcomes and health system resources needs under different policy implementation scenarios	Stock and flow model with transmission rates that vary by HIV infection stage and ART status	–
Martin et al. (2011)	Country - UK	HCV	Antiviral treatment among injecting drug users	National	Assess optimal treatment strategy for different economic and policy objectives	Frequency-dependent deterministic compartmental model (susceptible, chronically infected, treated)	Cost analysis
McKay et al. (2018)	Local - US	HIV	HIV counselling	Service	Describe the relationship between HR, intervention delivery and health outcomes by simulating different HR availability scenarios and observing effects on the other variables	Agent-based simulation	–
Peak et al. (2020)	Country – not specified	SARS-CoV-2	Epidemic preparedness	National	Compare effectiveness of individual quarantine and active monitoring at reducing effective reproductive number to below 1, under different feasibility scenarios	Density-dependent deterministic compartmental model (bSIS, recovery rate mediated by resources availability)	–
Putthasri et al. (2009)	Country - Thailand	Influenza	Modest pandemic mitigation	Decentralised	Define and quantify pandemic preparedness resources at the provincial level and estimate gaps under different scenarios	Density-dependent deterministic compartmental model	–
Rudge et al. (2012)	Regional - South-East Asia	Influenza	Epidemic preparedness	Decentralised	Estimate and compare resource gaps and their potential consequences in six countries	Density-dependent deterministic compartmental model (SEAIR)	–
Salomon et al. (2006)	Global	TB	Introduction of short-course regiments using new drugs	National	Examine the expected benefits of shorter drug regimens	Deterministic compartmental model (SIR-like model with treatment compartments)	–
Sébille and Valleron (1997)	Global	Nosocomial bacterial infection	Staff handwashing compliance to prevent transmission from patient contacts	Service	Develop a simulation of resistant pathogens spread in the hospital unit	Agent-based simulation	–
Shattock et al. (2016)	Country - Zambia	HIV	Multiple (model guides priority setting across the HIV cascade)	National	Assess time-varying optimal resource allocations for fixed and variable annual budgets and for various time horizons for measuring outcomes	Frequency-dependent deterministic compartmental model	–
Shim et al. (2011)	Country - US	Influenza	Seasonal influenza vaccination	National	Investigate age-dependent optimal vaccine distribution against influenza H1N1 influenza from the individual and population perspectives	Density-dependent deterministic compartmental model (SLIR)	Cost analysis
Stenberg et al. (2017)	Global	Health-related SDG targets	Multiple - 187 interventions targeting health-related SDGs and health systems strengthening	National	Estimate resource needs for strengthening health systems to reach universal health coverage in the SDG era	One Health tool, incorporating the interlinked epidemiological reference models for various disease areas (AIM, TIME, LiST)	Cost analysis
Stopard et al. (2019)	Country – provinces across Tanzania (Benin, South Africa limited implementation)	HIV	Multiple - behavioural change communication, pre-exposure prophylaxis, voluntary medical male circumcision and universal test-and-treat services	National	To investigate the impact of ‘real-world’ constraints on the resource allocation and possible health gains nationally	Frequency-dependent deterministic compartmental model	Optimisation
Verma et al. (2020)	Country – India	SARS-CoV-2	Treatment	National	Forecast need for hospital resources and assess surge capacity of health system	Density-dependent deterministic compartmental model (modified SEIR model with age-specific mixing patterns)	–
Zhang et al. (2020)	Country – not specified	Generic epidemic outbreak	Vaccination	National	Assess optimal vaccination policy in a resource-limited environment	Density dependent deterministic compartmental model (SIR with vaccination compartment)	–

AIM: AIDS Impact Model; AIDS: Acquired Immunodeficiency Syndrome; ART: Anti-Retroviral Therapy; CEA: Cost-Effectiveness Analysis; FTE: Full-Time Equivalent; HCV: Hepatitis C Virrus; HR: Human Resources; ICU: Intensive Care Unit; LiST: Lives Saved Tool; QALY: Quality-Adjusted Life-Years; SDG: Sustainable Development Goals.

### 3.1 Model structures

The majority of included studies used deterministic compartmental models of disease transmission, as shown in Table 1. However, all mathematical model structures commonly used to characterise the epidemiology of disease transmission were represented in the review, including agent-based simulations and stochastic models. Choice of model structure was determined by the characteristics of the disease, intervention and setting under study, rather than by the characteristics and objectives of the constrained analysis. For example, agent-based models were best suited for investigating nosocomial pathogen transmission (Ferrer et al., 2014; Sébille and Valleron, 1997), while stochastic models were used for cohort analyses assessing the impact of eradication campaigns (Marks et al., 2015) or measures to contain SARS-Cov-2 outbreaks (Peak et al., 2020). The structural decision may have been different if the focus had been the constrained analysis. For example, a compartmental model where the compartments reflect different levels of the health system in addition to disease progression and transmission could improve the analysis of human resource constraints. More details on the model structures represented are provided in the Supplementary File 1C.

### 3.2 Health system constraints and policy objectives

The types of health system constraints considered in the models and the objectives of the constrained analyses are described in Table 2. These ranged from constraints on service delivery inputs, mostly human resources and supplies, but also capital constraints such as equipment and hospital beds, to constraints on the demand for services (e.g. vaccine hesitancy) and other constraints on decision-making that affect the resource allocation process.

**Table 2:** Constrained analyses characteristics.

Lead author (year)	Constrained analysis objective	Non-financial constraints	Constraints identification	Constraints parametrisation and data sources	Approach for modelling constraints	Constraints implementation, details	Scenarios description
Adisasmito et al. (2015)	Feasibility assessment - produce realistic intervention impact estimates given health system constraints	HR, bed space, equipment, pharmaceutical supplies	Literature	Literature and secondary data analysis (AsiaFluCap survey)	Transmission model-based estimation - Calculate resource requirements	Transmission model linked to resource calculator to estimate requirements during outbreak. Model calculates depletion rate of resources based on average requirements to treat one case, estimated through a mix of data from literature and routine sources. Needs are compared to capacity, estimated through a survey administered as part of AsiaFluCap project	Two scenarios with different hospitalization and mortality rates
Alistar et al. (2013)	Efficient resource allocation - maximising impact given health system constraints	Political constraint on decision-making	Assumption	Assumption	Transmission model-based estimation - Limit effects and calculate costs along the cascade	REACH is an Excel-based user-friendly model helping policy makers allocate resources across different HIV control interventions. It comprises transmission dynamics and optimisation function. Optimisation done under budget constraint only, but political/social/ethical constraints on allocation of resources can be specified in the user interface. Outputs sheet includes estimates of health care resources needed to support the allocations	–
Anderson et al. (2014, 2018)	Feasibility assessment and efficient resource allocation - produce realistic intervention impact estimates and maximise impact given health system constraints	Political constraint on decision-making, demand side barriers to access	Assumption	Assumption	Transmission model-based estimation - Limit effects and calculate costs along the cascade	Constraints determine the way funds are allocated to key populations (MSM, other men, FSW, other women), geographical areas and throughout 5-year funding cycles (fully flexible, frontloaded, constant or back-loaded). Intervention choice optimised under the different resulting budget constraints. Constraints to implementation also parametrised in the form of uptake limits to certain intervention components	For key populations and districts (paper 1), all possible intervention scenarios compared by constructing health production functions for a given cost. For spending cycle (paper 2), 5 scenarios: 2 with complete spending flexibility (one of which with intervention change at 10 years), choices optimised over 30-year period; 3 with front-loaded, equal and back-loaded funding cycles, respectively, and choices optimised over each 5-year cycle
Bärnighausen et al. (2016)	Feasibility assessment - produce realistic intervention impact estimates given health system constraints	HR	Assumption	Literature	Transmission model-based estimation - Limit effects and calculate resource requirements along the cascade	Given current HR supply, number of patients treated is computed assuming fixed ratios for each cadre to patient. Model projects the impact of reallocating scarce HR to varying patient distributions in the different HIV disease stages and can estimate potential shortages	200 scenarios varying assumptions around HIV transmission probabilities, ART effect, retention and adherence. Two sets of constraints scenarios: one where allocation of HR is proportional to number of patients in TaSP and standard ART (treatment for advanced disease stages) pools, respectively; one where more HR allocated to pool with patients at more advanced disease stages
Barker et al. (2017)	Feasibility assessment - produce realistic intervention impact estimates given health system constraints	HR	Assumption	Secondary analysis of data from Tanzania and Mozambique on time spent by facility health workers delivering ART	Transmission model-based estimation - Calculate resource requirements	Model estimates total facility staff FTE needed for different ART differentiated care models, based on previous estimates of time spent delivering ART in Africa. An analysis of constraints is not presented because differentiated care models are expected to lead to cost and HR savings	–
Bottcher et al. (2015)	Feasibility assessment - produce realistic intervention impact estimates given health system constraints	Political constraint on decision-making, recurrent supplies	Assumption	Assumption	Transmission model-based estimation - Limit intervention effects	Model projects a global budget that increases by one unit with each additional healthy individual per unit of time and partially constrains recovery when available budget is insufficient for covering 'costs of healing'	–
Bozzani et al. (2018, 2020), Sumner et al. (2019)	Feasibility assessment and efficient resource allocation - produce realistic intervention impact estimates and maximise impact given health system constraints	HR, diagnostic equipment	Expert opinion	Secondary data collection from routine sources including district health information system (DHIS) and other Department of Health and Nursing Council records	Transmission model-based estimation - Limit effects and calculate resource requirements along the cascade	Unit costs and staff FTE to deliver different services are attached to model outputs to limit intervention effects once threshold of available resources is exceeded. Diagnostic constraint parametrised as maximum ratio of tests to TB notifications. Costs of 'relaxing' the constraints to achieve target coverage is calculated.	3 scenarios (least limiting, medium and most limiting) considered for each constraint (budget, diagnostic and HR), respectively, based on projections of future resource availability
Chen et al. (2019)	Feasibility assessment - produce realistic intervention impact estimates given health system constraints	Resources that are necessary to contain an epidemic (not specified)	Assumption	Assumption	Transmission model-based estimation - Limit intervention effects	A value R_c_, representing the level of resources in the system, is identified, whereby the epidemic can be effectively contained. If R < R_c_ the disease becomes widespread, recovery rate varies with time depending on average amount of resources that each infected individual receives	Scenarios explored with different levels of health system resourcing
Cruz-Aponte et al. (2011)	Feasibility assessment - produce realistic intervention impact estimates given health system constraints	Vaccine stockouts	Assumption	Assumption	Transmission model-based estimation - Limit intervention effects	Vaccine administration limited by daily maximum number. Vaccination campaign ends a) after some prescribed duration of time; or b) when stockpile is depleted. Results are compared with those from alternative model that ends campaign when target proportion of population is vaccinated.	Three scenarios varying the number of vaccines administered in a time period (56-, 28-, and 3-day campaign with different daily administration limits)
Curran et al. (2016)	Efficient resource allocation - maximising impact given health system constraints	HR, supplies and infrastructure	Group model building - System dynamics modelling techniques	Assumption	Transmission and system dynamics models linkage - Limit effects system-wide	The paper outlines possible ways of integrating transmission dynamics modelling with data generated from population surveys and sentinel surveillance and with system dynamics models to predict resource capacity during epidemic outbreaks and assist with resource allocation based on predicted pathogen spread	Multiple scenarios with varying disease transmission rates and health system capacity can be analysed
Dalgiç et al. (2017)	Efficient resource allocation - maximising impact given health system constraints	Vaccine stockouts	Assumption	Assumption	Constrained optimisation - Limit intervention effects	Optimise vaccine allocation in different age groups subject to constrained availability. Different objectives (minimise total costs, total infections, total deaths, total years of life lost)	Several vaccine coverage and delayed response time scenarios
Ferrer et al. (2014)	Feasibility assessment - produce realistic intervention impact estimates given health system constraints	HR	Assumption	Primary data collection at 5 ICUs on bed occupancy and staffing conditions	Transmission model-based estimation - Limit intervention effects	Model includes estimates of nurses' contact time with patients, which has an effect on pathogen spread. Daily rate of nurse absenteeism varied to adopt a fixed value between 10−40% and different coping mechanisms modelled	Systematic analysis of pathogen dissemination under different scenarios of pathogens circulating, level of nurses shortage and shortage management strategy
Hecht and Gandhi (2008)	Feasibility assessment - produce realistic intervention impact estimates given health system constraints	Political constraint on decision-making, demand side barriers to access	Literature and expert opinion	Assumptions based on expert consultation	Transmission model-based estimation - Limit intervention effects	Global demand for vaccine forecast by adding up demand estimates for individual country profiles	Four vaccine profile scenarios based on variations in efficacy, duration of protection and cost
Hontelez et al. (2016)	Efficient resource allocation - maximising impact given health system constraints	HR, infrastructure, demand-side barriers to access	Assumption	Assumptions made on effects of constraints on ART coverage. Costs of one-off investment needed to relax constraints calculated from routine AIDS spending reports	Transmission model-based estimation - Limit effects and calculate costs system-wide	Model calculates total investment needs, population health gains and cost-effectiveness of scaling-up new ART eligibility guidelines, including removal of health system constraints	Scenarios reflecting pessimistic, realistic and optimistic future health system developments, in which constraints apply to different extents
Krumkamp et al. (2011)	Efficient resource allocation - maximising impact given health system constraints	HR, pharmaceuticals supplies and other consumables	Assumption	Expert opinion and primary data collection (AsiaFluCap survey)	Transmission model-based estimation - Limit effects and calculate resource requirements along the cascade	Model constrains epidemic containment based on availability of resources and calculates resource depletion per hospital case. Resource usage data and impact of constraints estimated from a mix of survey data and expert opinion	Different epidemic control strategies modelled (antivirals stockpiling for critical cases, contact reductions)
Langley et al. (2014), Lin et al. (2011)	Efficient resource allocation - maximising impact given health system constraints	HR, diagnostic pathway bottlenecks, demand-side barriers to access	Group model building - Operational modelling techniques	Primary data collected from two diagnostic centres in Tanzania and calibrated using National TB programme reports	Transmission and operational models linkage - Limit intervention effects	Operational model outputs used to parametrise transmission model and vice versa. Operational component uses discrete-event simulation approach to model patient and sputum sample pathways	Different diagnostic algorithms modelled
Marks et al. (2017)	Feasibility assessment - produce realistic intervention impact estimates given health system constraints	Demand-side barriers to access	Assumption	Assumption	Transmission model-based estimation - Limit intervention effects	Eradication modelled under a range of plausible targeted treatment coverage estimates (65 %–95 %). Mass treatment compliance modelled as a random non-systematic process where every patient has the same, independent likelihood of receiving treatment	3 transmission scenarios modelled (low, medium, high) based on literature and expert opinion
Martin et al. (2015a, b)	Feasibility assessment - produce realistic intervention impact estimates given health system constraints	Implementation' constraints, demand-side barriers to access	Group model building - System dynamics modelling techniques	Literature and expert opinion	Transmission and system dynamics models linkage - Limit intervention effects	Scenario analysis where the flow of patients along the HIV testing and care cascade is determined by different sets of assumptions regarding policy implementation. These were defined in consultation with experts and based on the literature, by developing a system dynamics model that assesses the impact and relationships of different policy components	3 policy 'implementation' scenarios (low, high, perfect) and 3 testing policy scenarios (annual, five-year and no repeat offer of testing) combined to generate 9 unique combinations of policy conditions in addition to the base case
Martin et al. (2011)	Efficient resource allocation - maximising impact given health system constraints	Political constraint on decision-making	Assumption	Assumption	Constrained optimisation - Limit effects and calculate costs along the cascade	Optimal treatment strategy for HCV is examined under different economic and policy objectives: 1) minimise costs and QALY loss; 2) minimise prevalence; 3) minimise costs and QALY loss while achieving 20 % time prevalence reduction; 4) minimise costs while achieving 20 % time prevalence reduction	Analysis is repeated for a combination of annual budget constraints and two HCV baseline prevalences (30 % and 45 %)
McKay et al. (2018)	Feasibility assessment - produce realistic intervention impact estimates given health system constraints	HR	Assumption	Model parametrised with trial and implementation studies data and informed by published organizational and intervention sustainability models	Transmission model-based estimation - Limit effects and calculate resource requirements along the cascade	Model predicts the level of preventive services a health agency can provide given different combinations of i) staff positions; ii) turnover rates; iii) timing in training.	N/A
Peak et al. (2020)	Feasibility assessment - produce realistic intervention impact estimates given health system constraints	Barriers to effective contact tracing and quarantine interventions, including untrained monitoring of symptoms	Assumption	Assumption	Transmission model-based estimation - Limit intervention effects	R0 is estimated based on the implementation of quarantine and active monitoring in high- vs low-feasibility settings	Analysis compares a high- (90 % contacts traced and quarantined or monitored, reducing infectiousness by up to 90 %) and a low-feasibility setting (delays in locating contacts, imperfect quarantine)
Putthasri et al. (2009)	Efficient resource allocation - maximising impact given health system constraints	HR, supplies and infrastructure	Expert opinion	Expert opinion	Transmission model-based estimation - Calculate resource requirements	Actual and projected resources per case multiplied by the number of case-patients estimated by previous modelling exercises under different scenarios. Resource gaps estimated at the provincial level	3 epidemic (human-to-human transmission) scenarios analysed, with specific numbers of index cases and contacts: 1) from case-patients to caregivers; 2) localised clusters; 3) transmission resulting in substantial number of cases
Rudge et al. (2012)	Feasibility assessment and efficient resource allocation - produce realistic intervention impact estimates and maximise impact given health system constraints	HR, bed space, equipment, pharmaceutical supplies	Multi-criteria decision analysis - Delphi consensus process with a panel of 24 experts integrated with literature review	Primary data collection at health facilities to enumerate available resources. Gaps estimated based on literature on resource needs	Transmission model-based estimation - Calculate resource requirements	Available quantities of resources estimated through a survey sent out to hospitals, district health offices and ministries of health. Additional model parameters describing clinical pathway of infected individuals, conditional upon availability of resources	Model runs: i) available resources; ii) unlimited resources (to calculate gaps and compare with availability data from survey)
Salomon et al. (2006)	Feasibility assessment - produce realistic intervention impact estimates given health system constraints	HR, infrastructure	Assumption	Assumption	Transmission model-based estimation - Limit intervention effects	Constraints not explicitly modelled, but scenarios are analysed where it is assumed that the intervention reduces constraints to case detection, thus improving case detection rates	Scenarios were modelled with varying assumptions about case detection coverage (including one where constraints are relaxed), cure rates and DOTS scale-up
Sébille and Valleron (1997)	Feasibility assessment - produce realistic intervention impact estimates given health system constraints	Pharmaceutical supplies, political constraint on decision-making	Assumption	Assumption	Transmission model-based estimation - Limit intervention effects	Scenarios with different risk of patient-to-staff transmission based on whether procurement of two essential antibiotics is simultaneous (both available), sequential (only one available at a given time, then the other) or a mix of the two	Software allows for different assumptions to be specified before running simulations (e.g. drug procurement policy, staff handwashing compliance)
Shattock et al. (2016)	Efficient resource allocation - maximising impact given health system constraints	Political constraint on decision-making	Assumption	Assumption	Transmission model-based estimation - Limit intervention effects	Time-varying optimization i.e. minimising objective function (cumulative HIV infections) associated with the budget allocation, such that: i) total programme spending equals a pre-defined budget (either constant, front-loaded etc.) at each time point; or ii) total spending across the optimisation period is equal to pre-defined budget, but total spending at each point is optimally determined	4 optimization scenarios illustrating policy decisions where time considerations matter: 1) optimal 10-years allocation assuming baseline budget is annually available with no constraints to programme-specific allocation; 2) as in 1, but programme-specific funding cannot vary by more than 30% compared to baseline; 3) as in 1, but annual optimal allocation determined based on implementation and ethical constraints; 4) optimal 5-years allocation but cumulative new infections assessed after 5, 10 or 15 years, again within constraints
Shim et al. (2011)	Efficient resource allocation - maximising impact given health system constraints	Demand-side barriers to access	Assumption	Assumption	Transmission model-based estimation - Limit intervention effects	Decision to vaccinate characterised as a game, where monetary payoff for different age groups is modelled based on different individual strategies as well as on the average behaviour of the population	Two strategies modelled to calculate payoff to vaccinated and non-vaccinated: Nash and utilitarian
Stenberg et al. (2017)	Efficient resource allocation - maximising impact given health system constraints	HR, infrastructure, demand-side barriers to access	Assumption	Assumption	Transmission model-based estimation - Calculate intervention costs	Tracer interventions identified for each of the relevant SDGs, then gap estimated between current provision and universal coverage and country-specific programme costs multiplied by this gap. Costs estimated from the One Health Tool and from the literature. Progress towards 2030 targets adjusted by level of 'strength' of the health system (conflict, vulnerable, low-income, lower middle-income, upper middle-income)	Two financial space scenarios in each country, reflecting uncertainty around health systems' absorption capacity: i) ambitious, strengthening system towards global benchmarks and expanding coverage of full service package to 95%; ii) progress, not all SDG targets met by 2030 but improvements can be achieved by scaling up services delivered through the lower platforms
Stopard et al. (2019)	Efficient resource allocation - incidence minimizing	Political constraints on decision making (earmarking, externally imposed targets, minimising change to current program)	Assumption	Assumption	Transmission model-based estimation - Calculate intervention costs and impact	Constraints are modelled through initial conditions in each scenario representing minimum coverage by subgroups within the transmission model	Four scenarios of real-world constraints: 1) earmarking, where the first intervention funded would be PrEP for heterosexual women (excluding FSWs); 2) targets, where 90 % of PLHIV must receive UTT; 3) minimising change, baseline allocation represents an allocation at national level; and 4) all constriants simultaneously
Verma et al. (2020)	Feasibility assessment - produce realistic intervention impact estimates given health system constraints	Hospital beds, ICU beds and mechanical ventilation equipment	Assumption	Secondary data	Transmission model-based estimation - Limit effects and calculate resource requirements along the cascade	Available capacity estimated from public records, including for private sector. Capacity needs calculated based on requirements per case and turnover times from the literature. Capacity requirements during surge are based on model projections under different lockdown scenarios. Surge capacity compared to available capacity to estimate gap.	Different lockdown/social distancing scenarios
Zhang et al. (2020)	Efficient resource allocation - maximising impact given health system constraints	Vaccines availability	Assumption	Assumption	Constrained optimisation - Limit intervention effects	Optimise allocation of limited vaccines in order to minimise the number of infections	N/A

AIDS: Acquired Immunodeficiency Syndrome; ART: Anti-Retroviral Therapy; FTE: Full-Time Equivalent; HCV: Hepatitis C Virrus; HR: Human Resources; ICU: Intensive Care Unit; QALY: Quality-Adjusted Life-Years; SDG: Sustainable Development Goals.

The majority of articles relied on assumptions for identifying the constraints that applied to the setting and programme area of interest (n = 25, 66 %) and for quantifying the extent to which the constraints impacted intervention effects (n = 21, 55 %). For constraints identification, other sources were stakeholder elicitation in the form of expert opinion (n = 5), system dynamics modelling (n = 3), the literature (n = 1) and multi-criteria decision analysis using Delphi consensus (n = 1). Finally, two articles by Lin, Langley and colleagues described an operational model of the TB diagnostic pathway in Tanzania to identify bottlenecks and shortages, which was ‘linked’ to a transmission model; i.e. the operational model generated estimates of programmatic variables such as prevalence of treatment default and number of diagnostic centre visits, that were then used to parametrise the transmission model (Langley et al., 2014; Lin et al., 2011). Those studies that relied on data collection for parametrising constraints impact mostly used secondary sources (n = 7) or a mix of primary data collection and routine sources or expert opinion (n = 6). For example, modelling done using the AsiaFluCap simulator identified the resources needed for pandemic influenza response through expert elicitation (Rudge et al., 2012) and estimated available quantities and resource use per patient in the study countries through a survey integrated with data from the published literature (Adisasmito et al., 2015; Krumkamp et al., 2011).

Non-financial constraints influencing health providers’ ability to deliver health services were considered in two thirds of included studies (n = 28) (Adisasmito et al., 2015; Alistar et al., 2013; Bärnighausen et al., 2016; Barker et al., 2017; Bottcher et al., 2015; Bozzani et al., 2018, 2020; Chen et al., 2019; Cruz-Aponte et al., 2011; Curran et al., 2016; Dalgiç et al., 2017; Ferrer et al., 2014; Krumkamp et al., 2011; Langley et al., 2014; Lin et al., 2011; Martin et al., 2011; McKay et al., 2018; Peak et al., 2020; Putthasri et al., 2009; Rudge et al., 2012; Salomon et al., 2006; Sébille and Valleron, 1997; Shattock et al., 2016; Stopard et al., 2019; Sumner et al., 2019; Verma et al., 2020; Zhang et al., 2020), while only two studies considered constraints to the demand for health services (Hecht and Gandhi, 2008; Shim et al., 2011), and six articles considered both demand- and supply-side factors (Anderson et al., 2014, 2018; Hontelez et al., 2016; Marks et al., 2017; Martin et al., 2015a, b; Stenberg et al., 2017). The models that exclusively include demand-side constraints both focus on vaccines: one study projected the public and private demand for an AIDS vaccine candidate under different vaccine characteristics (efficacy, duration of protection, price), performance (acceptability, compliance) and country-level profile scenarios (including political ability and motivation to implement HIV/AIDS prevention programmes) (Hecht and Gandhi, 2008); the second study subdivided model compartments based on individual decisions to vaccinate against seasonal influenza, to assess the effects of vaccine hesitancy on coverage and to derive optimal vaccine allocation across age groups under a Nash (own interest) versus a utilitarian strategy (optimal for the population) (Shim et al., 2011).

Most commonly, the analyses that focussed on limits to the supply of health services incorporated a combination of HR, capital, equipment (infrastructure, hospital beds, logistics, ventilators etc.) and supplies constraints (drugs, vaccines and diagnostic consumables). These physical input constraints are not explicitly defined in a limited number of the analyses. For example, the 3S Surge System model for outbreak capacity planning consists of broadly defined ‘staff, stuff and structure’ (Curran et al., 2016) while three studies talk about non-specific resources necessary for controlling the spread of an epidemic (Bottcher et al., 2015; Chen et al., 2019; Peak et al., 2020) and other analyses refer to generic ‘implementation’ constraints that reduce the achievable coverage of interventions (Hontelez et al., 2016; Martin et al., 2015a, b).

A second set of supply-side non-financial constraints groups are distal factors deriving from political and social values and practices that determine how budgets are allocated, what activities are considered feasible or acceptable, and broader societal policy objectives that the system can pursue. Examples of models that allow for considering these constraints include the Resource Allocation for Controlling HIV tool, which allows users to specify interventions that cannot be implemented due to social, political or ethical concerns, or that have to receive a minimum/maximum level of funding for historical or strategic reasons (Alistar et al., 2013); the Optima model, which lets users analyse different budget allocation scenarios (constant, front-loaded, rear-loaded or initially scaled-up/down then later scaled-down/up over the funding cycle) (Shattock et al., 2016); and model developed by Stopard and colleagues, that examines different constraints to the efficient allocation of resources for HIV prevention, including externally imposed targets or limited capacity to modify existing programmes (Stopard et al., 2019). One set of studies in this group considers policy constraints both on the funding cycle (varying the flexibility of spending and the time horizon over which choices are to be optimised) and on how funds are allocated across key populations and geographical areas (Anderson et al., 2014, 2018).

### 3.3 Modelling approaches integrating non-financial constraints

As shown in Table 2, the rationale for considering health system constraints in the modelling studies was two-fold, with studies seeking to do one, or a combination, of the following: i) carry out a *feasibility assessment*, by producing reaslistic estimates of intervention impact (and costs) given the constraints; ii) guide *efficient priority setting*, by allocating resources in a way that maximises intervention impact given the constraints. Following from these objectives, the analytical approaches for considering constraints in the modelling studies can be grouped into two categories. The first category includes constrained estimation exercises, where intervention implementation is modelled at the maximum attainable coverage given the constraints. Effects (and costs) are thus limited at the level of the specific intervention, the disease cascade or the health system as a whole (Bottcher et al., 2015; Chen et al., 2019; Cruz-Aponte et al., 2011; Ferrer et al., 2014; Hecht and Gandhi, 2008; Marks et al., 2017; Peak et al., 2020; Salomon et al., 2006; Sébille and Valleron, 1997; Shattock et al., 2016; Shim et al., 2011; Stopard et al., 2019; Zhang et al., 2020).

The second category is unconstrained estimation, where interventions are modelled at full coverage but the gap in current resources for reaching that coverage is quantified in monetary or physical units, such as staff full-time equivalent (FTE) (Adisasmito et al., 2015; Barker et al., 2017; Putthasri et al., 2009; Rudge et al., 2012; Stenberg et al., 2017; Verma et al., 2020). Some of the studies in the review adopted a combination of these approaches, calculating both constrained impact estimates and the costs or resource requirements for relaxing the constraints (Alistar et al., 2013; Anderson et al., 2014, 2018; Bärnighausen et al., 2016; Bozzani et al., 2018, 2020; Hontelez et al., 2016; Krumkamp et al., 2011; McKay et al., 2018; Sumner et al., 2019). For example, Bozzani, Sumner and colleagues presented an analysis of different TB screening and diagnosis algorithms in South Africa under several constraints scenarios limiting effects along the TB prevention and care cascade to varying degrees, then modelled the additional staff FTE and costs of purchasing extra quantities of diagnostic consumables required to relax the constraints and achieve target coverage, observing any differences in the cost-effectiveness ranking of the screening options with and without constraints (Bozzani et al., 2018, 2020; Sumner et al., 2019).

In practice, constrained and unconstrained model-based estimation was most commonly achieved by combining transmission model outputs with unit costs (to address financial constraints) and other input per unit estimates, such as nurse FTE per output, to calculate resource usage at different intervention coverage levels and any additional requirements to relax constraints (Adisasmito et al., 2015; Alistar et al., 2013; Anderson et al., 2018; Bärnighausen et al., 2016; Barker et al., 2017; Bottcher et al., 2015; Bozzani et al., 2018, 2020; Cruz-Aponte et al., 2011; Ferrer et al., 2014; Hecht and Gandhi, 2008; Hontelez et al., 2016; Krumkamp et al., 2011; Marks et al., 2017; McKay et al., 2018; Putthasri et al., 2009; Rudge et al., 2012; Salomon et al., 2006; Sébille and Valleron, 1997; Shattock et al., 2016; Shim et al., 2011; Stenberg et al., 2017; Sumner et al., 2019; Verma et al., 2020). For instance, the agent-based model by McKay et *al*. analysed the relationship between HIV outcomes and staffing levels at a health agency by simulating changes over time in the number of HR positions, turnover rates and length of time for training newly recruited staff, and observing the effect of this HR constraint on the effectiveness of a prevention intervention (McKay et al., 2018).

A related approach adopted to incorporate constraints was the ‘linkage’ of disease transmission models with health system models, such as system dynamics (Curran et al., 2016; Martin et al., 2015a, b) or operational models (Langley et al., 2014; Lin et al., 2011). In this approach, model-based estimation relied on the health system models to generate estimates of the impact of constraints on intervention effects, which were then used to parametrise the transmission models. As an example, Curran and colleagues illustrated possible ways of integrating transmission models with system dynamics models to regulate the flows impacting on infection dynamics based on system capacity (Curran et al., 2016).

The last approach to integrate constraints was optimisation under a constraint other than the available budget. This approach was followed by two studies that sought to prioritise among different strategies, one for flu vaccine allocation in different age groups and one for HCV treatment, under different policy objectives such as minimising total incidence/prevalence, total deaths or total utility losses (Dalgiç et al., 2017; Martin et al., 2011; Zhang et al., 2020).

## 4 Discussion and conclusions

Incorporating health system elements that influence the priority setting process for disease control interventions, either by limiting the pace and scale of implementation or by otherwise determining their feasibility (as in the case of political or ethical constraints), is an increasingly common practice in the modelling literature. The main objectives of the studies reviewed were to constrain mathematical model outputs to approximate real-world implementation and to guide efficient resource allocation in the presence of constraints. They thus generated priority setting evidence that is more functional to the country-level planning cycle, in contrast to the ‘perfect implementation’ evidence generated by trials, trial-based economic evaluations and traditional target-driven modelling exercises (Menzies et al., 2019; Mikkelsen et al., 2017). One key advantage of these constrained analyses is that, by comparing target and actual implementation, the models allow analysts to calculate the resources needed for ‘relaxing’ the constraints, thus providing policy-makers with a more accurate estimate of the value for money of investing in a given intervention implemented at full scale.

Although the characteristics of the interventions and of the relative constraints are context-specific, there were patterns across settings in this review. For example, there was no distinction between demand-side and supply-side constraints in terms of the policy questions asked, whether about real-world impact or efficient investments (or both), and of the model structures used to explore them. Disease areas were also equally represented across models and similar objectives were pursued, for instance, by a study using an agent-based simulation to explore the allocation of flu vaccines in the presence of physical stockouts and a study using a SIR-like model to assess antiviral treatment strategies under different policy objectives (Dalgiç et al., 2017; Martin et al., 2011).

Constraints incorporation was achieved in two main ways, both of which can be accommodated by all mathematical model types: (i) model-based estimation, whereby limitations to intervention coverage were applied on the basis of either demand- or supply-side factors; (ii) optimisation, under a non-financial constraint or policy objective. The former was the most common approach overall, while the latter can be exclusively applied in analyses seeking to guide efficient resouce allocation. Approaches for identifying the applicable constraints and quantifying the extent of their impact varied in terms of strength, from unspecified assumptions to primary data collection, for example for building an operational model, and to structured stakeholder elicitation methods such as for systems dynamics modelling. Model-based estimation approaches thus varied according to the constraints quantification methods, and the dynamic transmission models were parametrised either in standard ways, using primary or secondary data, or through ‘linkage’ with the health system models (operational or system dynamics). The examples of model linkage in our sample are all from studies assessing interventions involving policy changes, such as a new HIV testing and linkage to care model, that are amenable to distal constraints more easily identified and quantified through group model building exercises involving a wide range of stakeholders.

This review builds on previous theoretical work on conceptualising and operationalising constraints (Vassall et al., 2016), but does not attempt to define the *feasibility* decision criterion. This concept and its influence on priority setting have been ill-defined in the literature and may encompass a range of aspects such as affordability, physical constraints that directly restrict access to services or technologies and arbitrary beliefs held by decision-makers and the wider environment that limit implementation in some way (Guindo et al., 2012; Tromp and Baltussen, 2012). In this review, the focus was restricted to non-financial constraints but the definition of constraints was kept deliberatly broad to capture all relevant incorporation approaches. The search strategy returned a number of records dealing with political, social and ethical constraints on the decision-making process, since it contained keywords around priority setting and decision-making criteria. We therefore introduced a working distinction between constraints on physical inputs and political constraints, including policy objectives. This latter category could, for example, include principles such as equity in cases where this objective is treated in the analysis as a *de facto* constraint to the roll-out or scale-up of an intervention, as in the study assessing the effects of prioritising key populations when delivering combination HIV prevention in Kenya (Anderson et al., 2014).

In conclusion, this review has shown that the inclusion of non-financial health system constraints in mathematical model-based priority setting can be accommodated within all model structures that are commonly used in epidemiological analyses. Despite the additional complexity, the enhanced models produce valuable information, including estimates of the costs of relaxing the constraints i.e. the true cost of the intervention at scale. As modelling techniques become more sophisticated and user-friendly and data availability improves, it will become increasingly possible to parametrise the models using real-time surveillance data, thus making the identification and quantification of constraints more viable and making models more locally-relevant and accessible for decision-makers within the policy timeframe (Alistar et al., 2013; Masoodian and Luz, 2017). However, further research is needed to categorise health system constraints, to assist their systematic operationalisation in models.

## Author contributions

**Fiammetta Bozzani**: Conceptualization, Methodology, Investigation, Formal Analysis, Writing – Oiginal draft preparation. **Gabriela Gomez**: Funding acquisition, Conceptualization, Supervision, Writing – Review and editing. **Anna Vassall**: Conceptualization, Supervision, Writing – Review and editing

## Funding

This work was funded by the 10.13039/100000865Bill and Melinda Gates Foundation through the TB Modelling and Analysis Consortium (TB-MAC, grant number OPP1135288).

## Declaration of Competing Interest

None. GBG is currently employed by Sanofi Pasteur as Regional Lead for vaccine epidemiology and modelling in Europe. Sanofi Pasteur has not provided funding for this work. GBG’s contribution to this work pertains to PhD supervisory activities as honorary staff at LSHTM.
