# Supplementary material for: Building resource constraints and feasibility considerations in mathematical models for infectious disease: A systematic literature review
Source: Epidemics. 2021 Jun;35:100450. doi: 10.1016/j.epidem.2021.100450 (PMC8207450; doi:10.1016/j.epidem.2021.100450)
Supplement: Supplementary file 1 [file mmc1.docx]

## Bozzani FM, Gomez GB, Vassall A. Building resource constraints and feasibility considerations in mathematical models for infectious disease: A systematic literature review

## Appendix A. Parametrising constraints in mathematical models, an example

Figure 1A illustrates the model transitions affected by human resource constraints along the tuberculosis care cascade (dashed lines).

Figure 1A. Example of modelling constraints impact on intervention effects


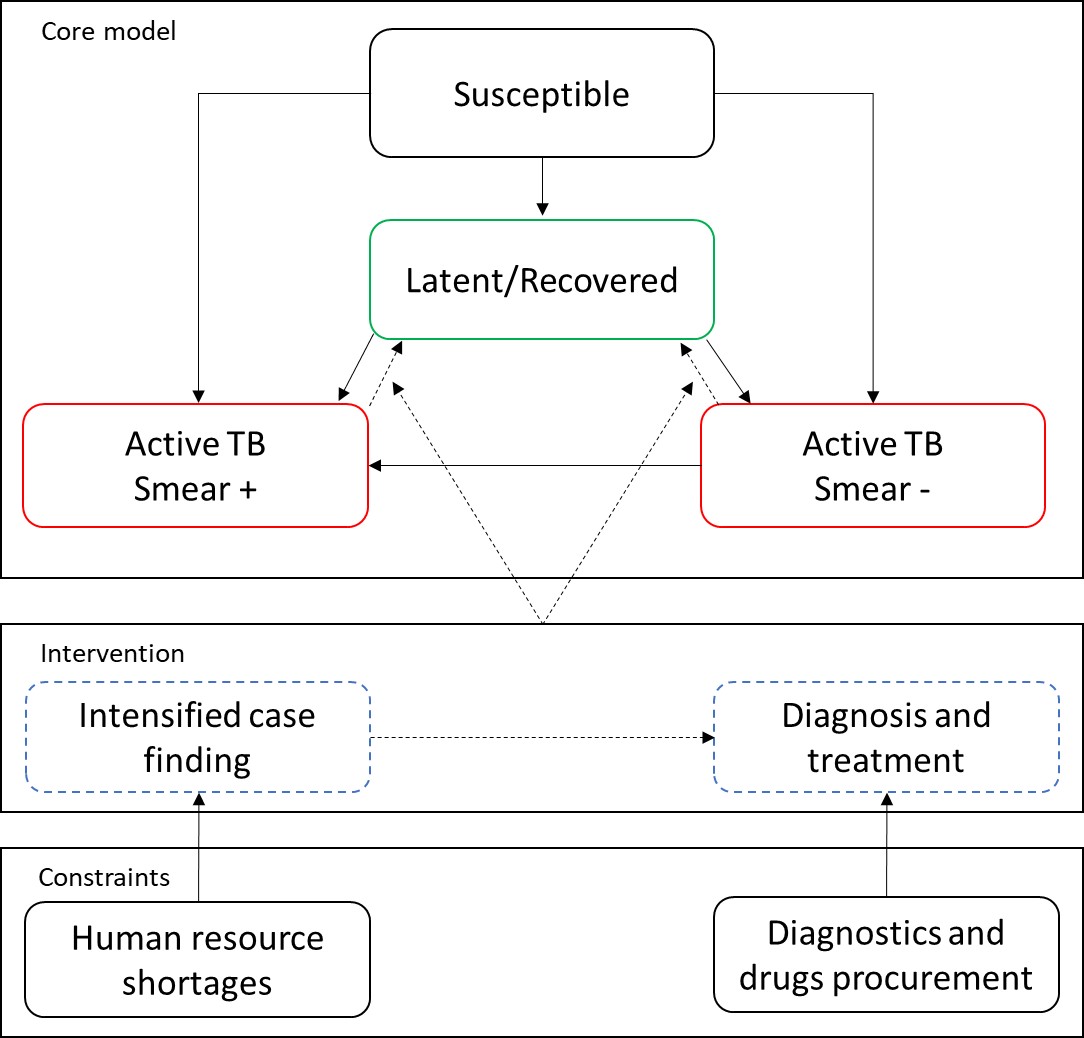


Adapted from (Houben et al., 2016). Dashed lines represent transitions impacted by constraints

## Appendix B. Literature search filters and results

### **Medline and Embase**

Medline and Embase were searched via OvidSP on 9^th^ May 2019. The search was updated on 9^th^ November 2020, to include all records published since the original search. Search results are summarised below (in brackets, results with limits applied: full text, humans)

|  | **Medline** | **Embase** |
| --- | --- | --- |
| *Search terms for infectious disease* |  |  |
| 1. exp Infectious Disease Transmission/ | 70,882 (10,670) | 48,356 (30,128) |
| 1. (infection or (infectious disease*) or outbreak or vaccin* or immuni#ation or (human immunodeficiency virus) or (HIV) or tuberculosis or (TB) or (antimicrobial resistance) or (AMR) or malaria or dengue or (mosquito-transmitted) or (mosquito-borne) or cholera or ebola or (hepatitis A) or (hepatitis B) or varicella or rubella or meningococc* or pneumococc* or influenza or (respiratory syndrome) or (SARS) or (h?emorragic fever) or (human papilloma virus) or (HPV) or chlamydia).ti,ab. | 1,835,247 (327,181) | 2,722,116 (379,371) |
| 1. or/1-2 | 1,863,174 (307,816) | 2,740,461 (346,632) |
| *Search terms for health systems research* |  |  |
| 1. exp Public Health Systems Research/ | 36 (4) | 85 (8) |
| 1. exp Systems Analysis/ | 89,479 (8,702) | 22,687 (1,212) |
| 1. (((health system*) and (building block*)) or ((health system*) adj3 research) or HPSR).ti,ab. | 922 (152) | 1,363 (196) |
| 1. ((health system* adj3 constraint*) or (resource* constraint*) or (resource* adj2 gap*) or (resource* adj1 need) or (demand adj3 constraint*) or (supply adj3 constraint*) or (implementation adj3 constraint*) or ((scal* up) and constraint*) or (capacity constraint*) or ((staffing or (human resource*) or HR or time) and (constraint* or shortage*))).ti,ab. | 24,262 (3,292) | 42,580 (5,265) |
| 1. (((feasib* or unfeasib*) adj3 intervention*) or (intervention adj3 (feasib* or unfeasib*))).ti,ab. | 3,162 (639) | 5,806 (1,077) |
| 1. Or/4-8 | 117,266 (12,709) | 72,204 (7,723) |
| *Search terms for mathematical modelling, economic evaluation and priority setting* |  |  |
| 1. exp Theoretical Models/ | 1,788,166 (196,565) | 10,463 (4,798) |
| 1. exp Economic Models/ | 15,261 (2,422) | 5,406 (230) |
| 1. exp Decision Support Techniques/ | 77,974 (10,735) | 26,393 (3,494) |
| 1. ((mathematic* or simulation or dynamic* or compartment* or (agent-based) or systems* or stochastic or deterministic or epidemic or epidemiologic* or transmission or cost*) and (model* or modeling* or modelling*)).ti,ab. | 534,555 (60,627) | 853,142 (79,899) |
| 1. ((decision-mak*) or (decision adj3 criteria) or (priorit* or (priority-setting))).ti,ab. | 219,849 (35,767) | 365,451 (53,550) |
| 1. ((economic evaluation) or (cost-effectiveness) or (cost-benefit) or (cost-utility) or (benefit-cost) or (cost-minimi#ation) or (health technology assessment) or (HTA)).ti,ab. | 67,767 (12,638) | 118,778 (17,176) |
| 1. (multi-criteria adj1 decision adj1 analysis).ti,ab. | 308 (32) | 626 (38) |
| 1. Or/10-17 | 2,351,214 (277,229) | 1,459,721 (146,532) |
| 1. 3 and 9 and 18 | **2,259 (461)** | **985 (215)** |

**Scopus**

*Search terms for infectious disease*

1. TITLE-ABS-KEY (“Infectious Disease Transmission”) *(10,742)*
2. TITLE-ABS-KEY (infection or (infectious disease*) or outbreak or vaccin* or immuni?ation or (“human immunodeficiency virus”) or (HIV) or tuberculosis or (TB) or (“antimicrobial resistance”) or (AMR) or malaria or dengue or (“mosquito-transmitted”) or (“mosquito-borne”) or cholera or ebola or (“hepatitis A”) or (“hepatitis B”) or varicella or rubella or meningococc* or pneumococc* or influenza or (“respiratory syndrome”) or (SARS) or (h*morragic fever) or (“human papilloma virus”) or (HPV) or chlamydia) *(3,974,834)*
3. #1 OR #2 *(3,974,834)*

*Search terms for health systems research*

1. TITLE-ABS-KEY (“Public Health Systems Research”) *(153)*
2. TITLE-ABS-KEY (“Systems Analysis”) *(169,032)*
3. TITLE-ABS-KEY ((“health system*” and “building block*”) or (“health system*” W/3 research) or HPSR) *(1,968)*
4. TITLE-ABS-KEY ((“health system*” W/3 constraint*) or “resource* constraint*” or (resource* W/2 gap*) or (resource* W/1 need) or (demand W/3 constraint*) or (supply W/3 constraint*) or (implementation W/3 constraint*) or (“scal* up” and constraint*) or “capacity constraint*” or “time constraint” or ((staffing or “human resource*” or HR) and (constraint* or shortage*))) *(65,508)*
5. TITLE-ABS-KEY (((feasib* or unfeasib*) W/3 intervention*) or (intervention W/3 (feasib* or unfeasib*))) *(6,494)*
6. #4 OR #5 OR #6 OR #7 OR #8 *(247,157)*

*Search terms for mathematical modelling, economic evaluation and priority setting*

1. TITLE-ABS-KEY (“Theoretical Models”) *(252,432)*
2. TITLE-ABS-KEY (“Economic Models”) *(20,385)*
3. TITLE-ABS-KEY (“Decision Support Techniques”) *(19,186)*
4. TITLE-ABS-KEY ((mathematic* or simulation or dynamic* or compartment* or (“agent-based”) or systems* or stochastic or deterministic or epidemic or epidemiologic* or transmission or cost*) and (model* or modeling* or modelling*)) *(6,212,320)*
5. TITLE-ABS-KEY ((decision-mak*) or (decision W/3 criteria) or (priorit* or (“priority-setting”))) *(11,001,863)*
6. TITLE-ABS-KEY ((“economic evaluation”) OR (“cost effectiveness”) OR (“cost benefit”) OR (“cost utility”) OR (“benefit cost”) OR (“cost minimi?ation”) OR (“health technology assessment”) OR (“HTA”)) *(487,891)*
7. TITLE-ABS-KEY (“multi criteria decision analysis”) *(3,349)*
8. #10 OR #11 OR #12 OR #13 OR #14 OR #15 OR #16 OR #17 *(16,962,390)*
9. #3 AND #9 AND #17 (**2,602**)

## Appendix C. Details on the structure of mathematical models analysed

This section aims to describe the mathematical model structures used to incorporate health system constraints in more detail. The focus is on influenza and HIV models, as the disease areas most represented in the review and presenting the widest variety of modelling approaches.

The majority of studies captured in this review used deterministic compartmental models of disease transmission. Models of influenza and SARS-Cov-2 transmission typically presented a SIR (susceptible, infected, recovered) or SEIR/SEAIR (as for SIR, but with explicit compartments for exposed and asymptomatic individuals) structure (Adisasmito et al., 2015, Curran et al., 2016, Krumkamp et al., 2011, Putthasri et al., 2009, Rudge et al., 2012), with the addition of a vaccination compartment where required by the analysis (Cruz-Aponte et al., 2011, Dalgiç et al., 2017, Shim et al., 2011). One analysis of influenza vaccine allocation aimed to compare the results of the deterministic compartmental model with those generated using agent-based simulation, to assess whether the strategies prioritised by the two models were different in any practical scenarios (Dalgiç et al., 2017). One influenza outbreak model presented a SIS structure (susceptible, infected, susceptible), where the rate at which individuals recovered and became susceptible again freed up resources, and the level of resources in the system in turn influenced the rate of recovery (Bottcher et al., 2015). Two models of nosocomial pathogens spread used agent-based simulation: one study analysing the impact of measures to address nurse shortages in the intensive care unit (ICU) ran 500-day simulations parametrised with data from a sample of ICUs in France (Ferrer et al., 2014); while another study used a Monte Carlo simulation model of the interactions between different patient and facility staff profiles (Sébille and Valleron, 1997).

All TB transmission models presented a SIR structure, with or without an additional compartment for latent infection and further stratified by other relevant characteristics including HIV and smear microscopy status as well as drug resistance (Bozzani et al., 2018, Bozzani et al., 2020, Sumner et al., 2019, Langley et al., 2014, Lin et al., 2011, Salomon et al., 2006). The majority of HIV transmission modles in the sample were also compartmental, with the most common structure distinguishing between age- and sex-stratified infected and uninfected compartments further subdivided by any relevant risk groups (Alistar et al., 2013, Anderson et al., 2014, Anderson et al., 2018, Bärnighausen et al., 2016, Stopard et al., 2019, Shattock et al., 2016). Two studies, one assessing differentiated antiretroviral therapy (ART) models and one looking at interventions targeting all health-related sustainable development goals (SDGs), generated projections using the AIDS Impact Model (AIM), which automatically aggregates transmission rates across all risk groups in the population and does not produce stratified outputs (Barker et al., 2017), and other models in the user-friendly interface Spectrum suite of software (Stenberg et al., 2017). One study aimed to build a linear predictive model of the demand for an HIV vaccine candidate in different countries based on its acceptability, parametrised as a function of vaccine efficacy and duration of protection as well as other local characteristics that determined the level of demand in different target populations (Hecht and Gandhi, 2008). Lastly, two papers analysing the effects of a new HIV testing and care policy in New York state, US, utilised a stock and flow model where transmission rates varied across different ‘HIV stage’ stocks, determined by CD4 counts derived from the literature; the model was then calibrated to local data and the transmission rates were used as fixed parameters in the stock and flow model, which is a system of integral and differential equations solved in a continuous, rather than a discrete simulation (Martin et al., 2015a, Martin et al., 2015b).

With regards to other disease areas, a model of hepatitis C virus (HCV) transmission among injecting drug users presented a SIR structure (Martin et al., 2011), while a stochastic Markov model of community transmission of yaws treated each disease stage as a discrete compartment, with infection rates dependent on transmission probability and number of infectious individuals (Marks et al., 2017).
